# Evaluation of Large Language Models for Emergency Triage in Urology: A Multi-Model Study

**DOI:** 10.3390/diagnostics16213510

**Published:** 2026-10-27

**Authors:** Qi Cai, Kaipeng Bi, Xinyu Zhang, Zhoujie Ye, Guanqiu Chen, Zheng Wang, Ziyan An, Weijun Fu

**Affiliations:** 1 Senior Department of Urology, Chinese PLA General Hospital, Beijing 100039, China; 2 China School of Medicine, Nankai University, Tianjin 300071, China; 3 Chinese PLA Medical School, Beijing 100853, China; 4 Department of Urology, Beijing Shijitan Hospital, Capital Medical University, Beijing 100038, China

**Keywords:** large language models, urologic emergencies, triage, artificial intelligence, emergency department

## Abstract

**Objective:** To evaluate the triage accuracy, response quality, safety, and repeatability of widely used large language models (LLMs) in urologic emergencies and to examine their potential clinical utility and risks. **Methods:** We developed 50 standardized clinical vignettes covering 10 categories of urologic emergencies, including testicular torsion, acute urinary retention, and Fournier gangrene. The set comprised high-risk vignettes requiring immediate emergency care, borderline vignettes, and atypical vignettes containing misleading cues. Six commercially available LLMs—Kimi 3.0, DeepSeek V4, Qwen 3.8-MAX, GPT-5.6 Sol (ChatGPT), Claude Opus 4.8, and Ant Afu (AQ)—were evaluated in Chinese-language interactions. Each vignette was submitted three times in independent conversations. Two urologists with more than 10 years of clinical experience assessed triage accuracy using a three-level reference standard (immediate emergency department [ED] evaluation, routine outpatient evaluation, or home observation). They also rated response quality across eight dimensions using a 5-point Likert scale and evaluated medical-error severity (0–3), response stability, and Chinese readability. Overall differences were assessed using the Friedman test, followed by pairwise Wilcoxon signed-rank tests with Holm adjustment. **Results:** A total of 900 responses were generated. Overall three-class accuracy was 89.11%, and the macro-averaged F1 score was 85.73%. Qwen achieved the highest accuracy (94.00%), whereas Claude achieved the lowest (80.67%). However, none of the pairwise comparisons between models reached statistical significance after Holm adjustment. All models generally recognized scenarios requiring immediate ED evaluation, but their ability to distinguish routine outpatient evaluation from home observation varied substantially. Mean content-quality scores ranged from 3.68 to 4.15; AQ achieved the highest overall score (4.15 ± 0.49), whereas DeepSeek achieved the lowest (3.68 ± 0.48). Clinically relevant errors occurred in 12.3% of responses, including major errors in 6.2% and dangerous errors in 3.1%. Three-run exact triage agreement ranged from 78% to 96%. Readability performance varied by metric, with no single model consistently outperforming the others. **Conclusions:** The six LLMs demonstrated some ability to perform three-level triage of urologic emergencies, supporting further investigation of their potential clinical applications. Nevertheless, important limitations remained in the management of borderline cases, response stability, and safety; some responses included potentially dangerous self-management instructions or could delay care for high-risk patients. Integration with authoritative guidelines, structured risk warnings, and human review will be necessary to improve clinical safety and reliability.

## 1. Introduction

Urologic emergencies are characterized by sudden onset and a need for prompt intervention; inappropriate self-management or delayed presentation may result in irreversible injury or even death [1]. Czajkowski et al. reported a substantial increase in the burden of acute urologic disease over a 10-year period, placing considerable pressure on healthcare systems [2].

A systematic review found that approximately 35–75% of individuals use the internet to obtain health-related information before consulting a healthcare professional [3]. Large language models (LLMs) can integrate symptom descriptions in natural language, generate differential diagnoses, and recommend care pathways, and they have rapidly entered patient health-information and clinical decision-support settings. Previous studies have shown that general-purpose LLMs encode substantial clinical knowledge, perform well on medical question-answering tasks, and can generate responses with favorable information quality and empathy [4,5]. However, answering knowledge questions correctly does not necessarily translate into correct clinical decisions. In emergency care, an inappropriate recommended course of action may directly lead to delayed treatment, unnecessary resource use, or unsafe self-management. Recent research on clinical decision-making has also identified systematic limitations when LLMs are tested beyond standardized questions or are presented with misleading or incomplete information [6].

Existing urologic research on LLMs has primarily focused on knowledge-based questions, patient education, or single-turn case consultations. Evaluation metrics have generally emphasized accuracy, completeness, and readability, whereas the ability of these models to determine urgency, identify potentially high-risk patients, and avoid inappropriate triage in emergency settings remains insufficiently studied [7,8]. We therefore developed standardized vignettes spanning several common urologic emergencies, including both typical presentations and atypical stress-test scenarios, and repeatedly evaluated multiple widely used LLMs to characterize their potential role and principal safety risks in preliminary triage of urologic emergencies.

## 2. Materials and Methods

The scenario set drew on two sources: topics related to urologic emergencies addressed in European Association of Urology guidelines and questions commonly posed by patients to healthcare professionals on social media platforms and websites. Each question was fully de-identified, and the patient’s age, past medical history, and the wording of the symptom descriptions were modified. The final set comprised 50 vignettes covering 10 categories of urologic emergencies: testicular torsion, acute urinary retention, Fournier gangrene/necrotizing fasciitis, paraphimosis, gross hematuria with clot retention, obstructive pyelonephritis/urosepsis, ischemic priapism, acute renal colic, suspected urethral injury, and testicular rupture/severe scrotal trauma (Supplementary Material). For each category, we included both high-risk vignettes requiring immediate ED evaluation and borderline vignettes not requiring immediate ED evaluation, thereby reducing the likelihood that models could respond solely on the basis of a disease label or a single keyword. Selected vignettes also included misleading cues, such as atypical or reassuring wording, patient minimization of symptoms, erroneous anchoring introduced by family members or prior experiences, changes in information order, and borderline clinical features.

We evaluated six commercially available LLMs: Kimi 3.0, DeepSeek V4, Qwen 3.8-MAX, GPT-5.6 Sol (ChatGPT), Claude Opus 4.8, and Ant Afu (AQ). All interactions were conducted in Chinese. For AQ, the evaluation was restricted to the function of its web interface and did not encompass the other functions of the broader AI health service. All interactions were conducted through the models’ web interfaces using default interface settings. Each vignette was submitted verbatim as the sole user message in a new conversation, without any additional task instruction or follow-up prompt. All requests were submitted from Beijing on 15 August 2026. Each submission was initiated in a new conversation, and chat history was cleared to prevent information carryover. To assess response stability, the order of the vignettes was fully randomized, and each vignette was submitted independently three times to every model.

### 2.1. Quality Assessment

Two urologists with more than 10 years of clinical experience independently evaluated the standardized question-and-answer texts. First, the 50 vignettes were assigned to one of three reference triage levels: immediate ED evaluation, defined as a recommendation to attend the ED immediately or as soon as possible; routine outpatient evaluation, defined as a recommendation to seek medical attention when the symptoms did not require immediate ED care; or home observation, defined as self-care or observation being appropriate on the basis of the information provided [9,10]. Disagreements regarding the reference levels were adjudicated by a third expert, and the reference standard was finalized before response evaluation. The two urologists subsequently independently classified each model response into the same three triage categories. Disagreements regarding response classification were also adjudicated by the third expert. Second, the 900 model responses were rated in random order across eight dimensions: D1, identification of the target condition or a reasonable nonemergent explanation; D2, identification and description of relevant features of the target condition; D3, appropriate differential diagnosis; D4, accurate explanation of why immediate ED evaluation was or was not required; D5, appropriate timing and care pathway; D6, correct precautions or contraindications that would not delay care; D7, scenario-relevant escalation triggers in nonemergency responses; and D8, information prioritization and actionability. Each applicable dimension was rated on a five-point Likert scale: 1, strongly disagree; 2, disagree; 3, neither agree nor disagree; 4, agree; and 5, strongly agree [11]. For each response, the two raters’ scores were first averaged within each applicable dimension, after which the arithmetic mean across all applicable dimensions was calculated. Nonapplicable dimensions were excluded from both the numerator and denominator. The two experts also independently rated the severity of medical errors: 0, no error; 1, minor error not expected to alter management; 2, major error that could misdirect evaluation or the care pathway; and 3, dangerous error that could cause delay, harm, or unsafe self-management.

### 2.2. Stability Assessment

The three responses generated by the same model for the same vignette constituted one repeated-measures unit. A model-vignette unit was classified as fully consistent when all three responses assigned exactly the same triage category. The three-run exact agreement rate for a model was calculated as the proportion of fully consistent units among its 50 vignettes.

### 2.3. Readability Assessment

Because all model interactions were conducted in Chinese, readability was assessed as a secondary outcome using AlphaReadabilityChinese. This tool assesses text at the lexical, syntactic, and semantic levels using nine metrics.

Four metrics are negatively associated with readability, meaning that higher scores indicate greater reading difficulty: lexical richness, syntactic richness, noun semantic richness, and noun semantic noise. The other five metrics are positively associated with readability, meaning that higher scores indicate easier comprehension: noun semantic accuracy, verb semantic accuracy, joint noun-verb semantic accuracy, content-word semantic accuracy, and noun semantic clarity [12].

### 2.4. Statistical Analysis

Statistical analyses were performed using Python 3.13. We used the 50 scenarios as paired blocks for statistical analysis. The primary outcome was three-class triage accuracy, defined as the proportion of responses whose assigned triage category matched the reference standard. Overall differences among the six models were assessed using the Friedman test. Pairwise comparisons were conducted using paired Wilcoxon signed-rank tests with Holm adjustment for multiple comparisons. Cohen’s κ was used to assess interrater agreement before adjudication for triage classification and medical-error severity, as well as agreement between model triage classifications and the reference standard. Interrater agreement for the overall D1–D8 score was assessed using absolute-agreement intraclass correlation coefficients for a single rater, ICC(A,1), and the mean of two raters, ICC(A,2). Repeatability across the three runs was assessed using Fleiss’ κ. ED sensitivity was the proportion of reference-ED responses classified as immediate ED care, whereas non-ED specificity was the proportion of reference-non-ED responses classified as non-ED care. The worst-case undertriage rate was the proportion of the 25 reference-ED vignettes for which at least one of the three runs recommended non-ED care. A two-sided *p* < 0.05 was considered statistically significant. Ninety-five percent confidence intervals (CIs) for descriptive means were estimated using 5000 cluster-bootstrap resamples at the vignette level.

## 3. Results

**Figure 1:**
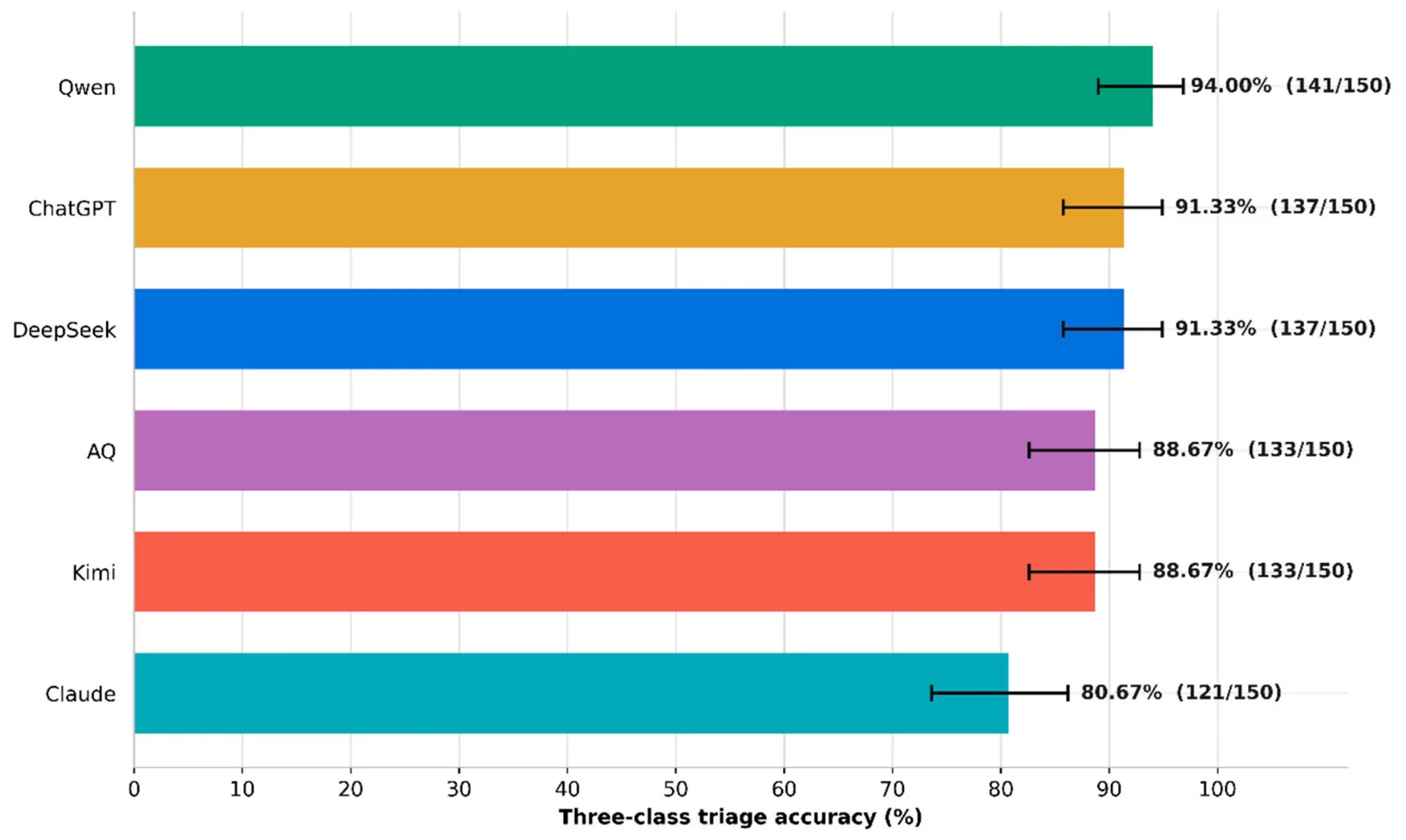
Three-Class Triage Accuracy by Large Language Model.

**Figure 2:**
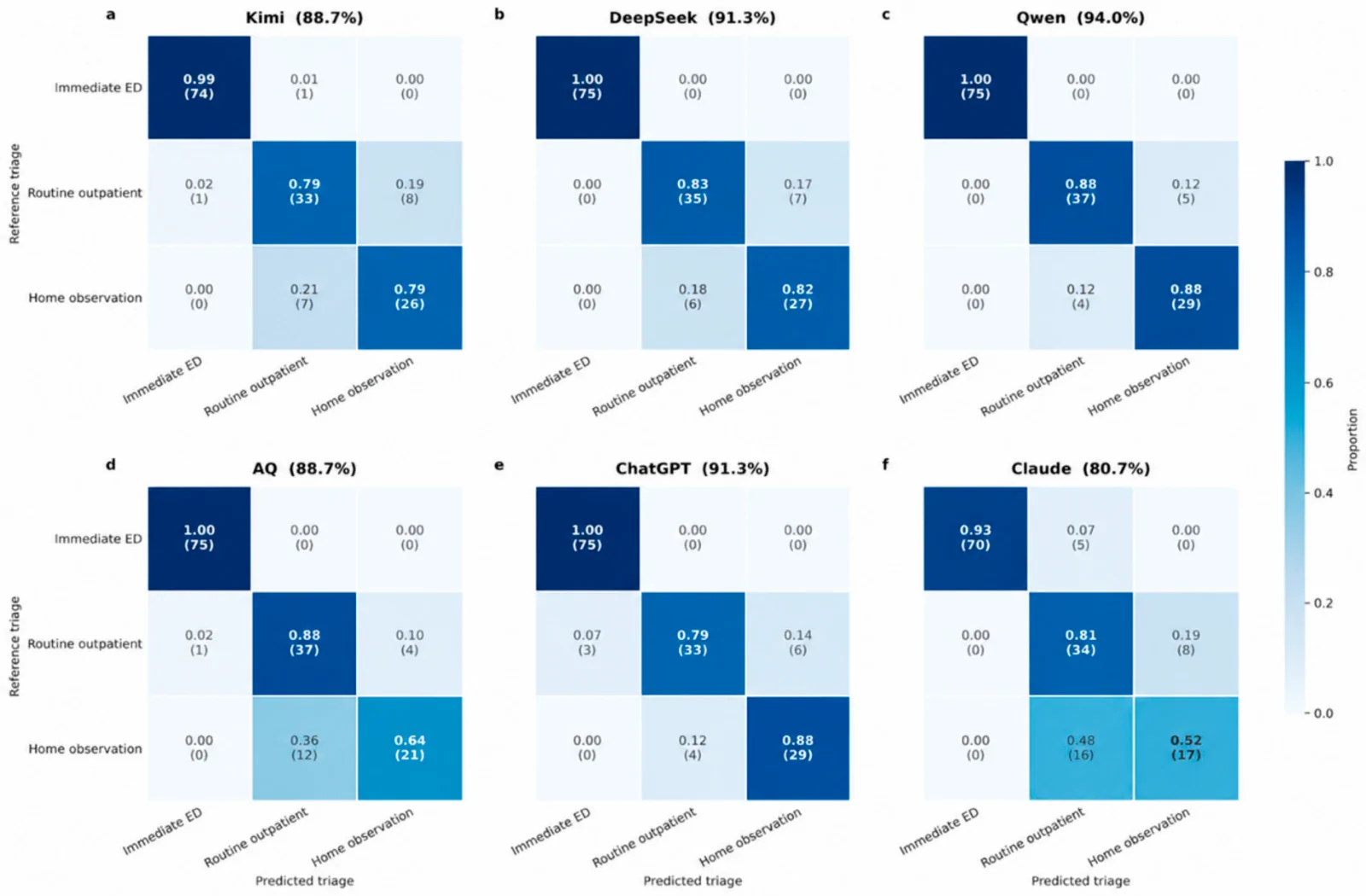
Confusion Matrices of Three-Level Triage Results by Large Language Model. (**a**) Kimi; (**b**) DeepSeek; (**c**) Qwen; (**d**) AQ; (**e**) ChatGPT; (**f**) Claude. ED, emergency department.

**Figure 3:**
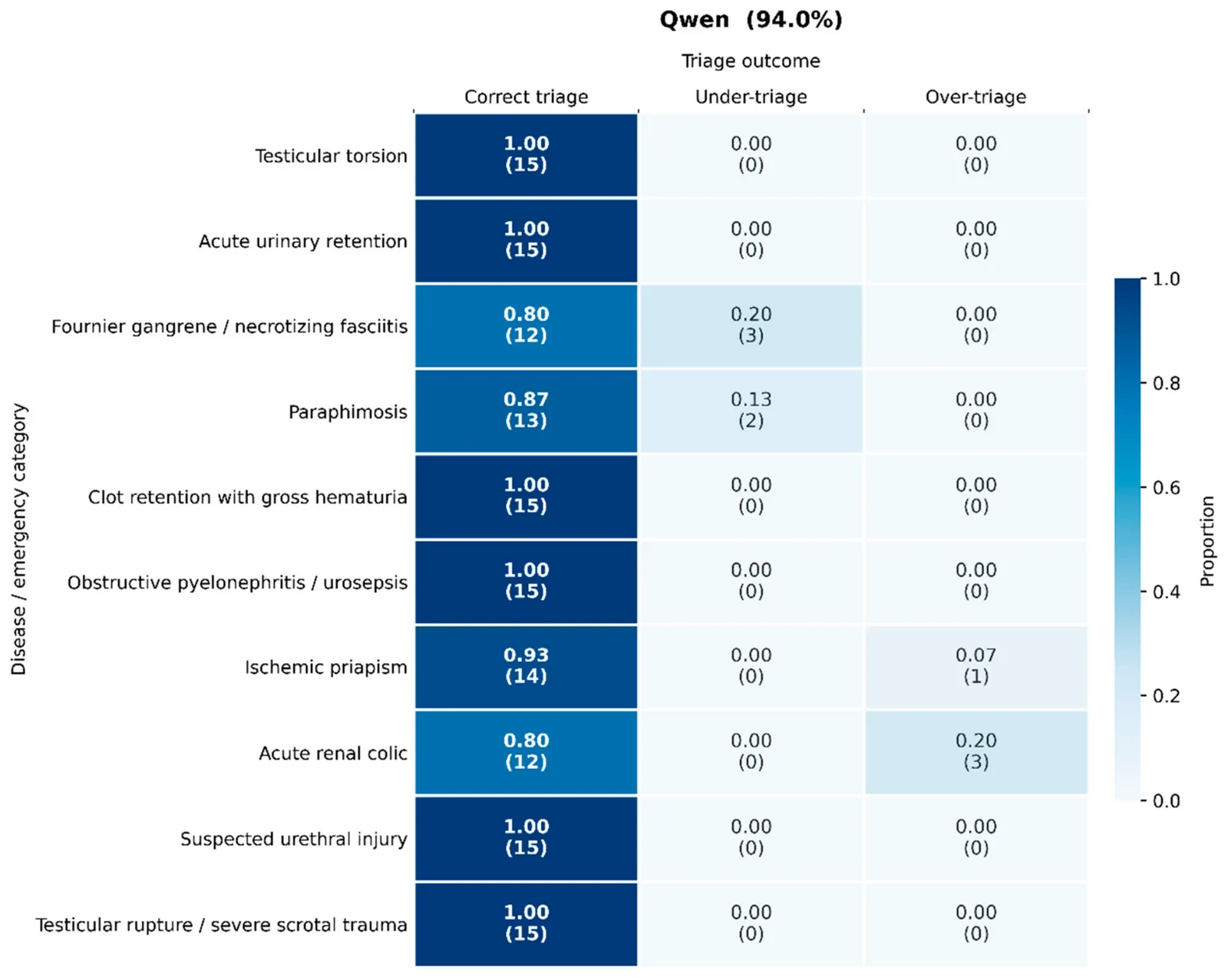
Qwen Triage Outcomes Across 10 Urologic Emergency Categories.

Each model generated 150 responses, yielding 900 responses in total. Interrater agreement for triage classification before adjudication was excellent (Cohen’s κ = 0.991; 95% CI, 0.983–0.998). Across the 50 vignettes, the three-run exact triage agreement rates were 84% for Kimi (Fleiss κ 0.83; 95% CI, 0.715–0.929), 90% for DeepSeek (Fleiss κ 0.89; 95% CI, 0.798–0.976), 92% for Qwen (Fleiss κ 0.92; 95% CI, 0.829–0.979), 96% for AQ (Fleiss κ 0.96; 95% CI, 0.888–0.991), 94% for ChatGPT (Fleiss κ 0.94; 95% CI, 0.856–0.988), and 78% for Claude (Fleiss κ 0.76; 95% CI, 0.640–0.876). Overall three-class accuracy was 89.11% (802/900; 95% CI, 83.00–94.44%), and the macro-averaged F1 score was 85.73% (Table 1). Among the six models, Qwen achieved the highest accuracy (94.00%) and Claude the lowest (80.67%); accuracy for the remaining models ranged from 88.67% to 91.33% (Figure 1). The omnibus difference among models was statistically significant (χ^2^ = 17.814, *p* = 0.0032); however, none of the 15 pairwise comparisons remained significant after Holm adjustment. The smallest adjusted *p* value was 0.058 for the comparison between Qwen and Claude. The confusion matrices showed generally strong recognition of scenarios requiring immediate ED evaluation (Figure 2). In the vignette-level worst-case analysis, at least one of the three runs recommended non-ED care for 1 of the 25 emergency vignettes with Kimi (4.0%) and 3 with Claude (12.0%). In the secondary binary analysis of immediate ED versus non-ED triage, ED sensitivity ranged from 93.33% to 100%. DeepSeek, Qwen, AQ, and ChatGPT achieved 100% sensitivity, compared with 98.67% for Kimi and 93.33% for Claude. Non-ED specificity was 100% for DeepSeek, Qwen, and Claude, 98.67% for Kimi and AQ, and 96.00% for ChatGPT. In contrast, the models varied substantially in distinguishing routine outpatient evaluation from home observation: class-specific recall ranged from 78.6% to 88.1% for routine outpatient evaluation and from 51.5% to 87.9% for home observation. Qwen showed the most balanced performance across the three triage categories. We therefore further examined its outcomes by emergency category. Qwen achieved 100% correct triage in six categories: testicular torsion, acute urinary retention, gross hematuria with clot retention, obstructive pyelonephritis/urosepsis, suspected urethral injury, and testicular rupture/severe scrotal trauma. Mis-triage nevertheless occurred in the other four categories (Figure 3).

**Figure 4:**
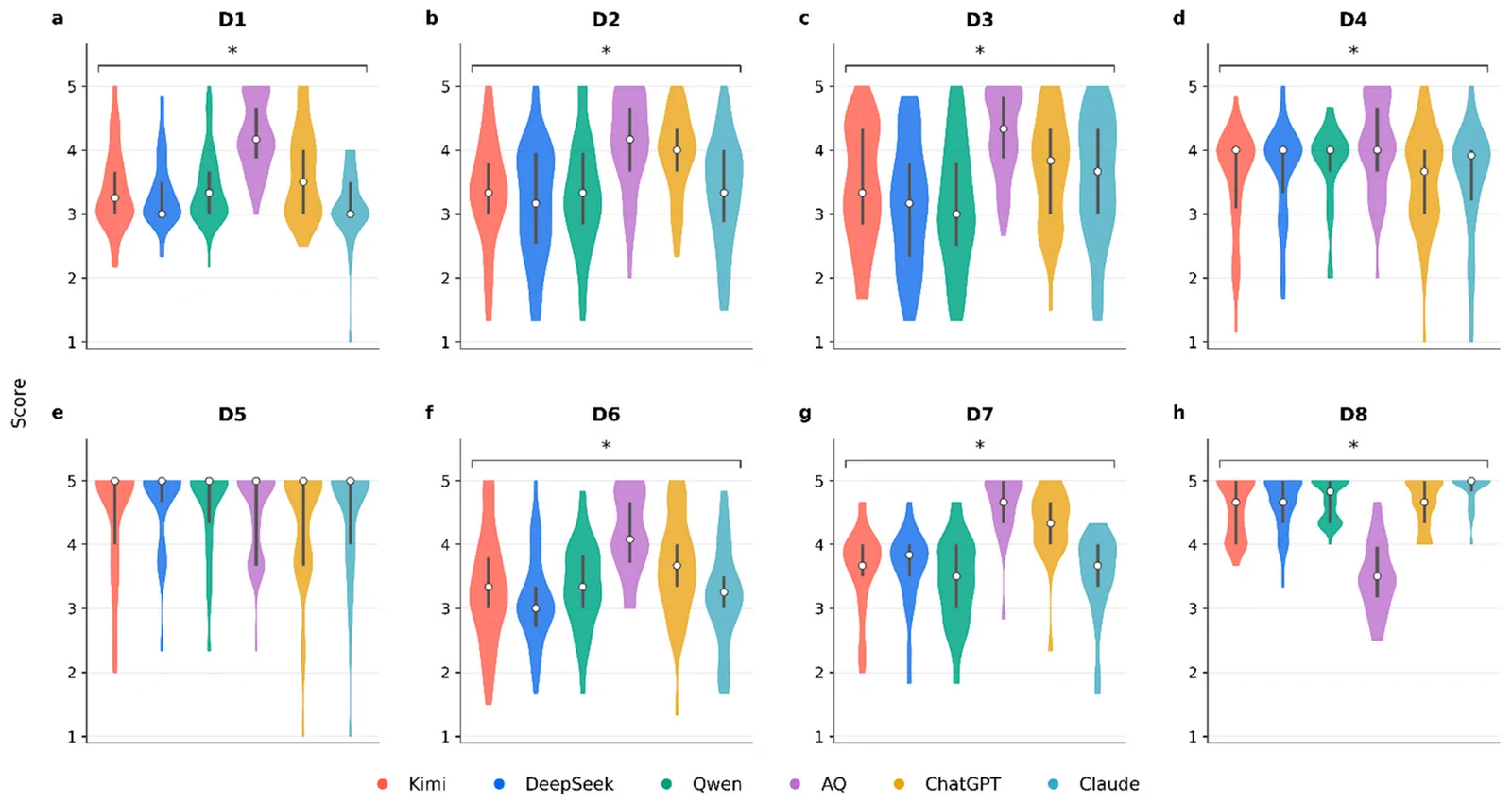
Content-Quality Assessment by Large Language Model. * *p* < 0.05.

Interrater reliability was excellent for the overall D1–D8 content quality score across 900 responses. The single-rater, absolute-agreement intraclass correlation coefficient was ICC(A,1) = 0.965 (95% CI: 0.955–0.971). For the mean of the two raters, which was used in the primary analysis, ICC(A,2) was 0.982 (95% CI: 0.977–0.986). Mean scores across the eight content-quality dimensions ranged from 3.68 to 4.15 (Table 2). AQ achieved the highest score (4.15 ± 0.49; 95% CI, 4.07–4.24), whereas DeepSeek achieved the lowest (3.68 ± 0.48; 95% CI, 3.57–3.79). The omnibus difference among models was statistically significant (χ^2^ = 86.69, *p* < 0.001). In pairwise comparisons, AQ had a higher overall content-quality score than each of the other five models, and ChatGPT scored higher than Kimi, DeepSeek, Qwen, and Claude; all corresponding Holm-adjusted *p* values were <0.01. Comparisons among Kimi, DeepSeek, Qwen, and Claude were not significant. At the dimension level, scores differed among models for all dimensions except D5 (*p* < 0.05). AQ achieved the highest scores for D1, D2, D3, D4, D6, and D7, whereas Claude performed best on D8 (information prioritization and actionability) (Figure 4).

Interrater agreement in the assessment of medical errors was high (weighted Cohen’s κ = 0.92; 95% CI, 0.87–0.96). Among the 900 responses, 789 (87.7%) contained no clinically relevant error, 27 (3.0%) contained a minor error, 56 (6.2%) contained a major error, and 28 (3.1%) contained a dangerous error. Across models, the rate of any error ranged from 10.7% to 14.7%, the rate of major or dangerous errors ranged from 4.7% to 12.0%, and the rate of dangerous errors ranged from 0.7% to 5.3% (Table 3). Omnibus differences among models were not significant for any error (*p* = 0.956), major or dangerous errors (*p* = 0.382), or dangerous errors (*p* = 0.886).

**Figure 5:**
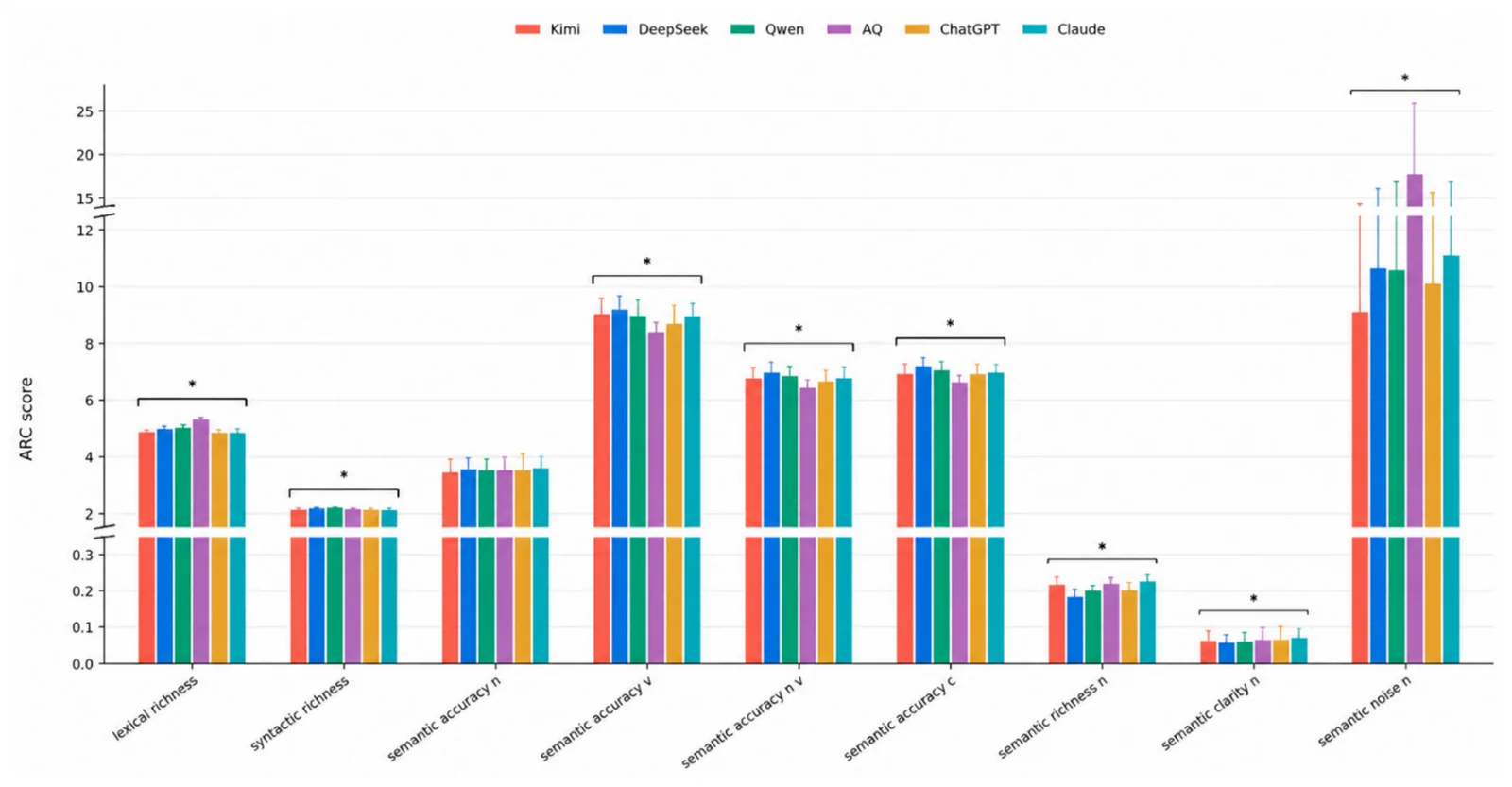
AlphaReadabilityChinese Readability Scores. n, nouns; v, verbs; c, content words. * *p* < 0.05.

In the secondary readability analysis, eight of the nine metrics differed significantly among models; noun semantic accuracy was the only exception (Table 4). ChatGPT had the lowest lexical richness. Kimi and Claude had the lowest syntactic richness, whereas Qwen had the highest. DeepSeek achieved the highest verb, joint noun-verb, and content-word semantic accuracy and the lowest noun semantic richness. AQ had the highest lexical richness and noun semantic noise and the lowest values for several semantic-accuracy metrics. Claude achieved the highest noun semantic clarity (Figure 5). Taken together, the metrics did not identify a single model as consistently the most readable.

## 4. Discussion

In recent years, the strong knowledge-integration and natural-language capabilities of LLMs have led to their increasing use in patient health consultation, medical education, and clinical decision support. Previous studies have shown that LLMs can perform history taking, formulate differential diagnoses, and provide management recommendations, with potential strengths in diagnostic accuracy, communication, and empathy [13,14]. In emergency triage, some studies have reported favorable recognition of high-acuity patients, but overall accuracy, repeatability, and handling of low-acuity and borderline cases remain suboptimal. Moreover, LLM clinical decisions may be influenced by information framing, prompt wording, and nonclinical cues; these systems are therefore not yet suitable for independent triage without professional oversight [15,16]. Against this background, we evaluated six LLMs in urologic-emergency triage across multiple domains, including triage accuracy, content quality, medical errors, repeatability, and Chinese readability.

Overall triage accuracy in this study was 89.11%. Scenarios requiring immediate ED evaluation were generally recognized well, whereas routine outpatient evaluation and home observation were more frequently confused. The observed accuracy should be interpreted in the context of the vignette distribution. Half of the vignettes (25/50) required immediate ED evaluation. A simple “always recommend immediate ED evaluation” rule would therefore achieve 50% overall accuracy and miss no emergencies, while incorrectly referring all 25 non-immediate-ED vignettes to the ED. This baseline illustrates why recognition of emergencies alone is insufficient to establish useful triage performance; overtriage and discrimination among all three care pathways must also be considered. Qwen achieved the highest accuracy and macro-averaged F1 score, whereas AQ achieved the highest composite D1-D8 content-quality score. Nevertheless, 12.3% of the 900 responses contained clinically relevant errors, and repeated responses from the same model to the same vignette were not always consistent. Franc et al. performed 10,980 queries using 61 Canadian Triage and Acuity Scale scenarios and reported an overall accuracy of 47.5%, an undertriage rate of 13.7%, and an overtriage rate of 38.7% [17]. In an evaluation of 39,375 ED records and seven LLMs, Nedos et al. reported that the best model achieved a raw accuracy of approximately 61.7%, with a maximum weighted kappa of only 0.467 [18]. Among actual ED patients, Colakca et al. reported an accuracy of 76.6% for five-level Emergency Severity Index triage by ChatGPT [15]. The overall accuracy in our study exceeded that reported in most previous general ED triage studies. This difference may reflect rapid advances in LLMs in recent years, but it may also be attributable to the narrower task design: our study was restricted to 10 categories of urologic emergencies, used a three-level classification directly linked to the recommended care setting, and provided relatively complete and consistently formatted vignette information. Fewer classes, a clearly defined specialty scope, and standardized inputs may all have reduced task difficulty. Sarbay et al. reported an F1 score of 0.821 for ChatGPT in Emergency Severity Index levels 1–2, compared with 0.461 across all cases [19]. Meral et al. found that GPT-4 achieved 70.60% overall triage accuracy and 85.11% accuracy for Emergency Severity Index levels 1–2; the corresponding values for Gemini were 59.70% and 87.77%, respectively [20]. These findings are consistent with the generally strong recognition of immediate-ED scenarios in our study and suggest that conditions with salient hallmark features—such as testicular torsion, urinary retention, urosepsis, and severe genitourinary trauma—may be more likely to trigger an ED recommendation. This does not, however, mean that models identify all high-risk states. In 960 structured stress tests, Ramaswamy et al. found that ChatGPT Health correctly recognized classic emergencies such as stroke and anaphylaxis but undertriaged 51.6% of gold-standard emergencies. When family members or friends minimized symptoms, recommendations for borderline cases changed significantly, with most shifts toward less urgent care [21]. LLMs may therefore perform well when an emergency is presented through prototypical labels or features yet struggle when urgency depends on clinical evolution, incomplete information, or misleading anchors.

Although the omnibus Friedman test indicated a difference among models, none of the 15 pairwise comparisons was statistically significant after adjustment. Qwen can therefore be described as having the highest accuracy and macro-averaged F1 score in this study, but the results do not establish its statistical superiority over the other models. In an evaluation spanning six biomedical task categories and 26 datasets, Jahan et al. found that no LLM consistently outperformed all others across tasks. A recent multilingual medical evaluation likewise showed that accuracy varied significantly according to the model, input language, prompt language, and response format. Collectively, these findings indicate that model performance is contingent on the task, prompt, language, version, and outcome [22,23]. During expert review, both urologists expressed concern about Claude’s triage recommendation for a vignette suggestive of early Fournier gangrene. The patient was 58 years old, had diabetes, and presented with perineal numbness and swelling, a low-grade fever, and no improvement with topical treatment—a high-risk presentation requiring immediate ED evaluation to exclude a necrotizing soft-tissue infection. Claude partly attributed the numbness to diabetic peripheral neuropathy and recommended a routine urology, colorectal surgery, or dermatology visit “today or tomorrow”, advising ED care only if the temperature exceeded 38.5 °C, the swelling spread, or substantial pain developed. Both experts judged that this response relied excessively on pain intensity and a fever threshold and could falsely reassure the patient that immediate ED care was unnecessary, thereby delaying diagnosis and debridement of Fournier gangrene. The 2026 EAU Guidelines on Urological Infections strongly recommend: “Commence repeated surgical debridement for Fournier’s gangrene within 24 h of presentation”. This time frame concerns surgical treatment after presentation and should not be interpreted as allowing patients with suspected Fournier’s gangrene to delay emergency evaluation. Thus, in a patient with diabetes who develops perineal swelling, sensory changes, and systemic signs of infection, routine outpatient follow-up should not replace immediate ED evaluation even when pain and fever are not pronounced [24,25]. Correct triage, moreover, does not guarantee that the remainder of a response is safe. In a vignette describing severe glans swelling, violaceous discoloration, marked pain, and paraphimosis of 4 h’ duration, Kimi and DeepSeek correctly recommended immediate ED evaluation but also provided detailed instructions for sustained glans compression and self-administered manual reduction. Both raters judged these instructions to be unsafe. Manual reduction in paraphimosis is a clinician-performed procedure that may require appropriate analgesia and a controlled technique [26]. In a case already showing signs of ischemia, detailed instructions for unsupervised reduction were considered unsafe because repeated unsuccessful attempts could delay definitive emergency treatment.

With respect to readability, AQ responses often included specialized terminology and diagnostic or therapeutic detail beyond what patients needed for an immediate care decision. For example, in a vignette involving suspected obstructive urinary tract infection in an older adult, AQ used terms such as “hemodynamic status,” “percutaneous nephrostomy,” and “ureteral stent.” In an ischemic priapism vignette, it further described “cavernosal blood gas analysis,” “intracavernosal injection of a sympathomimetic agent,” and “phenylephrine.” Although medically informative, extensive testing and treatment details may increase cognitive burden for patients deciding whether to seek immediate care and may reduce the salience of the core instruction to attend the ED immediately. This pattern may reflect AQ’s positioning as a specialized medical tool. Prompts that prespecify plain language and a structured response—for example, “First state in one simple sentence whether emergency care is needed, and then explain the most important reason”—may reduce comprehension demands and foreground the primary action recommendation [11].

From a translational perspective, whether LLMs can safely support patients’ care-seeking decisions requires further evaluation in prospective studies involving real patients. Future work should address three priorities. First, retrieval-augmented generation grounded in authoritative guidelines may reduce hallucinations and improve adherence to evidence. Second, calibrated confidence thresholds should trigger mandatory human review for low-confidence or borderline responses. Third, triage-specific regulatory standards should require not only accuracy testing before deployment but also adversarial robustness testing, repeatability testing, and systematic error-mode analysis [27,28,29].

This study systematically compared the performance of LLMs in urologic-emergency triage and assessed their potential risks. First, the study used only 50 simulated vignettes, some of which were clinically similar, limiting the diversity of the case set and its ability to reflect the complexity of real-world patient presentations. Second, all responses were generated using a fixed prompt in single-turn conversations; we did not evaluate the models’ ability to elicit additional history, identify distortions in patient descriptions, or manage multi-turn interactions. Third, content quality and safety were evaluated primarily by two urologists. Although the ratings were blinded, some subjectivity may remain. In addition, assigning equal weight to the D1–D8 dimensions may obscure critical safety errors in the composite score. Future multicenter prospective studies in actual patients should incorporate assessments by emergency physicians, triage nurses, and patients and should link model recommendations to care-seeking behavior and clinical outcomes to further establish the safety and effectiveness of LLM-assisted triage for urologic emergencies.

## 5. Conclusions

The six LLMs demonstrated some ability to perform three-level triage of urologic emergencies, supporting further investigation of their potential clinical applications. Qwen achieved the highest observed accuracy and macro-averaged F1 score, although no accuracy comparison remained statistically significant after Holm adjustment. AQ achieved the highest composite content-quality score. Integration with authoritative guidelines, structured risk warnings, and human review will be needed to further improve safety and clinical reliability.

## Figures and Tables

**Table 1:** Three-Class Triage Performance by Large Language Model.

Model	Accuracy	95% CI	Macro F1	Weighted Kappa	95% CI	Undertriage, n (%)	Overtriage, n (%)
Kimi	88.67%	81.33–95.33	85.27%	0.912	0.849–0.960	9 (6.0%)	8 (5.3%)
DeepSeek	91.33%	84.00–97.33	88.31%	0.933	0.875–0.978	7 (4.7%)	6 (4.0%)
Qwen	94.00%	88.00–98.67	91.91%	0.954	0.904–0.990	5 (3.3%)	4 (2.7%)
AQ	88.67%	79.33–96.00	84.36%	0.906	0.826–0.968	4 (2.7%)	13 (8.7%)
ChatGPT	91.33%	83.33–98.00	88.96%	0.934	0.866–0.982	6 (4.0%)	7 (4.7%)
Claude	80.67%	70.67–89.33	75.09%	0.837	0.747–0.907	13 (8.7%)	16 (10.7%)

Macro F1 = unweighted mean of the three class-specific F1 scores; undertriage = recommendation less urgent than the reference level; overtriage = recommendation more urgent than the reference level; CI = confidence interval.

**Table 2:** Content-Quality Scores by Large Language Model.

Model	D1	D2	D3	D4	D5	D6	D7	D8	Mean ± SD	95% CI
Kimi	3.39	3.33	3.44	3.56	4.43	3.32	3.63	4.56	3.72 ± 0.50	3.61–3.83
DeepSeek	3.32	3.19	3.14	3.68	4.61	3.14	3.63	4.65	3.68 ± 0.48	3.57–3.79
Qwen	3.42	3.30	3.14	3.77	4.57	3.36	3.41	4.71	3.74 ± 0.41	3.64–3.83
AQ	4.22	4.10	4.25	4.08	4.52	4.18	4.62	3.49	4.15 ± 0.49	4.07–4.24
ChatGPT	3.62	3.94	3.70	3.55	4.35	3.66	4.33	4.65	3.96 ± 0.53	3.86–4.06
Claude	3.14	3.35	3.54	3.53	4.48	3.19	3.52	4.86	3.72 ± 0.49	3.60–3.83

**Table 3:** Medical Errors by Large Language Model.

Model	No Error	Minor Error	Major Error	Dangerous Error	Any Error
Kimi	134 (89.3%)	1 (0.7%)	9 (6.0%)	6 (4.0%)	16 (10.7%)
DeepSeek	128 (85.3%)	7 (4.7%)	7 (4.7%)	8 (5.3%)	22 (14.7%)
Qwen	132 (88.0%)	11 (7.3%)	6 (4.0%)	1 (0.7%)	18 (12.0%)
AQ	133 (88.7%)	0	12 (8.0%)	5 (3.3%)	17 (11.3%)
ChatGPT	132 (88.0%)	0	15 (10.0%)	3 (2.0%)	18 (12.0%)
Claude	130 (86.7%)	8 (5.3%)	7 (4.7%)	5 (3.3%)	20 (13.3%)

**Table 4:** Readability Assessment Using AlphaReadabilityChinese.

Metric	Kimi	DeepSeek	Qwen	AQ	ChatGPT	Claude	*p* Value
Lexical richness	4.87	4.99	5.04	5.33	4.84	4.86	<0.001
Syntactic richness	2.12	2.18	2.20	2.14	2.14	2.12	<0.001
Noun semantic accuracy	3.46	3.56	3.53	3.54	3.54	3.59	0.144
Verb semantic accuracy	9.04	9.21	8.99	8.40	8.71	8.98	<0.001
Joint noun-verb semantic accuracy	6.76	6.98	6.86	6.43	6.66	6.77	<0.001
Content-word semantic accuracy	6.93	7.19	7.07	6.64	6.92	6.97	<0.001
Noun semantic richness	0.22	0.19	0.20	0.22	0.21	0.23	<0.001
Noun semantic clarity	0.06	0.06	0.06	0.07	0.07	0.07	<0.001
Noun semantic noise	9.13	10.65	10.58	17.74	10.13	11.13	<0.001

## Data Availability

The raw data supporting the conclusions of this article will be made available by the authors on request.

## Supplementary Materials

The following supporting information can be downloaded at: https://www.mdpi.com/article/10.3390/diagnostics16213510/s1, Supplementary Material: 50 vignettes.

## References

[1] Manjunath A.S., Hofer M.D. (2018). Urologic Emergencies. Med. Clin. N. Am..

[2] Czajkowski S., Lajkosz K., Koziarz A., Carr D., Finelli A., Lee J.Y. (2022). The rising burden of acute urological disease at an urban, academic hospital network. Can. Urol. Assoc. J..

[3] Mueller J., Jay C., Harper S., Davies A., Vega J., Todd C. (2017). Web Use for Symptom Appraisal of Physical Health Conditions: A Systematic Review. J. Med. Internet Res..

[4] Singhal K., Azizi S., Tu T., Mahdavi S.S., Wei J., Chung H.W., Scales N., Tanwani A., Cole-Lewis H., Pfohl S. (2023). Large language models encode clinical knowledge. Nature.

[5] Ayers J.W., Poliak A., Dredze M., Leas E.C., Zhu Z., Kelley J.B., Faix D.J., Goodman A.M., Longhurst C.A., Hogarth M. (2023). Comparing Physician and Artificial Intelligence Chatbot Responses to Patient Questions Posted to a Public Social Media Forum. JAMA Intern. Med..

[6] Hager P., Jungmann F., Holland R., Bhagat K., Hubrecht I., Knauer M., Vielhauer J., Makowski M., Braren R., Kaissis G. (2024). Evaluation and mitigation of the limitations of large language models in clinical decision-making. Nat. Med..

[7] Davis R., Eppler M., Ayo-Ajibola O., Loh-Doyle J.C., Nabhani J., Samplaski M., Gill I., Cacciamani G.E. (2023). Evaluating the Effectiveness of Artificial Intelligence-powered Large Language Models Application in Disseminating Appropriate and Readable Health Information in Urology. J. Urol..

[8] Song H., Xia Y., Luo Z., Liu H., Song Y., Zeng X., Li T., Zhong G., Li J., Chen M. (2023). Evaluating the Performance of Different Large Language Models on Health Consultation and Patient Education in Urolithiasis. J. Med. Syst..

[9] Kopka M., Feufel M.A., Berner E.S., Schmieding M.L. (2023). How suitable are clinical vignettes for the evaluation of symptom checker apps? A test theoretical perspective. Digit. Health.

[10] Semigran H.L., Linder J.A., Gidengil C., Mehrotra A. (2015). Evaluation of symptom checkers for self diagnosis and triage: Audit study. BMJ.

[11] Chen G., Bi K., Cai Q., Wang Z., An Z., Fu W. (2026). Comparative study on the efficacy of large language models (LLMs) in health management for patients with urological and andrological diseases: A multidimensional quality evaluation and applicability analysis. World J. Urol..

[12] Lei L., Wei Y., Liu K. (2024). AlphaReadabilityChinese: A tool for the measurement of readability in Chinese texts and its applications. Foreign Lang. Their Teach..

[13] Tu T., Schaekermann M., Palepu A., Saab K., Freyberg J., Tanno R., Wang A., Li B., Amin M., Cheng Y. (2025). Towards conversational diagnostic artificial intelligence. Nature.

[14] Alowais S.A., Alghamdi S.S., Alsuhebany N., Alqahtani T., Alshaya A.I., Almohareb S.N., Aldairem A., Alrashed M., Bin Saleh K., Badreldin H.A. (2023). Revolutionizing healthcare: The role of artificial intelligence in clinical practice. BMC Med. Educ..

[15] Colakca C., Ergın M., Ozensoy H.S., Sener A., Guru S., Ozhasenekler A. (2024). Emergency department triaging using ChatGPT based on emergency severity index principles: A cross-sectional study. Sci. Rep..

[16] Draelos R.L., Afreen S., Blasko B., Brazile T.L., Chase N., Desai D.P., Evert J., Gardner H.L., Herrmann L., House A.V. (2026). Large language models provide unsafe answers to patient-posed medical questions. npj Digit. Med..

[17] Franc J.M., Cheng L., Hart A., Hata R., Hertelendy A. (2024). Repeatability, reproducibility, and diagnostic accuracy of a commercial large language model (ChatGPT) to perform emergency department triage using the Canadian triage and acuity scale. Can. J. Emerg. Med..

[18] Nedos I., Zagalioti S.-C., Kofos C., Katsikidou T., Vellidou D., Astrinakis K., Karagiannis I., Giannakopoulos P., Michaloudi S., Apostolopoulou A. (2026). Is Artificial Intelligence Ready for Emergency Department Triage? A Retrospective Evaluation of Multiple Large Language Models in 39,375 Patients at a University Emergency Department. J. Clin. Med..

[19] Sarbay İ., Berikol G.B., Özturan İ.U. (2023). Performance of emergency triage prediction of an open access natural language processing based chatbot application (ChatGPT): A preliminary, scenario-based cross-sectional study. Turk. J. Emerg. Med..

[20] Meral G., Ateş S., Günay S., Öztürk A., Kuşdoğan M. (2024). Comparative analysis of ChatGPT, Gemini and emergency medicine specialist in ESI triage assessment. Am. J. Emerg. Med..

[21] Ramaswamy A., Tyagi A., Hugo H., Jiang J., Jayaraman P., Jangda M., Te A.E., Kaplan S.A., Lampert J., Freeman R. (2026). ChatGPT Health performance in a structured test of triage recommendations. Nat. Med..

[22] Jahan I., Laskar M.T.R., Peng C., Huang J.X. (2024). A comprehensive evaluation of large Language models on benchmark biomedical text processing tasks. Comput. Biol. Med..

[23] Strasser L.M., Anschuetz W., Dennstädt F., Hastings J. (2026). Performance Evaluation of Large Language Models in Multilingual Medical Multiple-Choice Questions: Mixed Methods Study. JMIR Med. Educ..

[24] Bonkat G., Kranz J., Cai T., Geerlings S.E., Köves B., Lambregts M.M.C., Mantica G., Pilatz A., Medina-Polo J., Schneidewind L. (2026). EAU guidelines on urological infections. Eur. Assoc. Urol..

[25] Puvanendran R., Huey J.C., Pasupathy S. (2009). Necrotizing fasciitis. Can. Fam. Physician.

[26] Hussein H. (2024). Clinical Procedure for the Management of Paraphimosis, Version 2.

[27] Busch F., Hoffmann L., Rueger C., van Dijk E.H., Kader R., Ortiz-Prado E., Makowski M.R., Saba L., Hadamitzky M., Kather J.N. (2025). Current applications and challenges in large language models for patient care: A systematic review. Commun. Med..

[28] Gallifant J., Afshar M., Ameen S., Aphinyanaphongs Y., Chen S., Cacciamani G., Demner-Fushman D., Dligach D., Daneshjou R., Fernandes C. (2025). The TRIPOD-LLM reporting guideline for studies using large language models. Nat. Med..

[29] Vasey B., Nagendran M., Campbell B., Clifton D.A., Collins G.S., Denaxas S., Denniston A.K., Faes L., Geerts B., Ibrahim M. (2022). Reporting guideline for the early-stage clinical evaluation of decision support systems driven by artificial intelligence: DECIDE-AI. Nat. Med..

