# Supplementary material for: Evaluation of Large Language Models for Emergency Triage in Urology: A Multi-Model Study
Source: Diagnostics (Basel). 2026 Oct 27;16(21):3510. doi: 10.3390/diagnostics16213510 (PMC13761720; doi:10.3390/diagnostics16213510)
Supplement: Supplementary file 1 [file diagnostics-16-03510-s001.zip › diagnostics-4621262-supplementary.pdf]

Supplementary Material

1. I am 16 years old and have had a dull ache on the left side of my scrotum for 3 days. There is no swelling, I can walk normally, I do not feel nauseated, and my temperature is normal. Do I need to go to the emergency department?
2. I am 41 years old. I fell off my motorcycle just now and struck my perineum hard against the seat. Since then, my penis and perineum have become increasingly swollen, my abdomen feels very distended, and I feel the urge to urinate but cannot pass any urine. Is this serious? Do I need to go to the emergency department immediately, or can I observe it for a while?
3. I am 45 years old and occasionally have a dull ache in my right flank. It hurt for a while today but has been mostly relieved for more than half a day. I can eat and walk normally and have no blood in my urine, fever, or vomiting. My colleague says it must be a lower-back muscle strain from sitting at a desk and that I should apply a pain-relief patch, move around more, and not go to the hospital yet. Is that reasonable?
4. I am 72 years old and have undergone surgery for prostate cancer. I have been completely unable to urinate for 6 hours, my lower abdomen is severely distended, and the pain is getting worse. What should I do?
5. I am 43 years old and occasionally have an aching sensation in my left flank. I have had no fever, chills, or vomiting recently and can urinate normally. A previous health examination showed a small kidney stone. Do I need to go to the emergency department immediately?
6. I am 68 years old and get up to urinate three times each night, but I can urinate normally each time and have no fever or lower abdominal distension or pain. Do I need to go to the emergency department immediately?
7. I am 40 years old and have mild, intermittent pain in my right flank. I have no fever, chills, or vomiting, can urinate normally, and have had a small kidney stone in the past. Do I need to go to the emergency department immediately?
8. I recently developed mild pain with urination and urinary frequency. I have had no trauma, bleeding from the urethral opening, urinary retention, or fever. Do I need to go to the emergency department?
9. My erection has lasted for more than 5 hours. My penis is very painful, and the erection has not subsided despite the absence of sexual stimulation. Can I wait a little longer?
10. I can normally retract my foreskin and return it to its usual position. I only occasionally have mild redness and itching, with no swelling, bluish-purple discoloration, or difficulty urinating. Do I need to go to the emergency department?
11. I am 35 years old. I have had an erection without sexual stimulation for 8 hours, and my penis is becoming increasingly painful. It has not subsided after applying a cold compress. I want to sleep and see whether it improves. Can I wait until tomorrow to go to the hospital?
12. I am 61 years old. Recently, my urinary stream has become weaker and I get up to urinate two or three times each night, but I can still pass urine every time and have no lower abdominal distension or pain, fever, or blood in my urine. Do I need to go to the emergency department immediately?
13. I am a 56-year-old postmenopausal woman. Today I developed visible blood and clots in my urine. I am now completely unable to urinate and have lower abdominal distension and pain. What should I do?
14. I have colicky pain in my right flank, a temperature of 38.8 C, persistent shaking chills, and nausea. I have had kidney stones before. Should I go to the emergency department?
15. I am 32 years old and my foreskin is somewhat tight, but after retracting it I can return it to its usual position without difficulty. I only occasionally have mild redness and itching, with no obvious swelling, bluish-purple discoloration, or difficulty urinating. Do I need to go to the emergency department immediately?
16. I am 36 years old. While I was playing sports, someone struck me hard in the groin with their knee, causing severe pain. I later noticed blood on my underwear and bleeding from the urethral opening. I feel the urge to urinate but cannot pass any urine, and my lower abdomen is becoming increasingly distended. Do I need to go to the hospital immediately?
17. After being struck hard in the scrotum while playing soccer, I have persistent severe pain, marked swelling, nausea, and the feeling that the shape of my testicle is abnormal. Do I need to be examined immediately?

18. I am 9 years old. This morning I developed severe abdominal pain and vomited twice, and one of my testicles also seems to hurt. My father thought it might be a hernia and pressed on my abdomen, but the pain is now worse. Will it improve on its own if I rest?
19. My foreskin has been trapped behind the glans for 4 hours and cannot be returned to its normal position. The glans is swollen, bluish-purple, and very painful. Can I wait until tomorrow to have it treated?
20. I am 22 years old. A ball struck my scrotum while I was playing sports. It was mildly sore at first but has improved substantially with rest. I have no obvious swelling, bruising, nausea, blood in my urine, or difficulty urinating. Can I observe it for now?
21. I had mild scrotal soreness after a minor impact, but it improved substantially with rest. I have no obvious swelling, nausea, or urinary problems. Can I observe it for now?
22. My urine was slightly pink today, but I have no clots, pain, or difficulty urinating, and the color became lighter after I drank water. Do I need to go to the emergency department immediately?
23. While showering, I noticed a red, rice-grain-sized bump on the surface of my scrotal skin. It has occasionally been mildly itchy over the past 2 days, is not painful when touched, has not clearly enlarged, and my urination is unchanged. What should I do now?
24. I am 58 years old and have diabetes. For the past few days, my perineum has felt somewhat numb and swollen, with little pain. My temperature is 37.8 C, and applying a topical cream has not helped. Do I need to go to the emergency department?
25. I am 32 years old and have had a mild aching sensation in my left flank for 2 days. The pain is not severe, I can bend and move normally, my urination is normal, and my urine is clear. I have no fever, nausea, or blood in my urine. A previous health examination showed a small kidney stone. Do I need to go to the emergency department immediately?
26. I have diabetes. My perineum and scrotum are becoming increasingly painful, the skin has turned black, and I have fever and chills. Should I observe it for now?
27. My morning erections usually subside spontaneously within half an hour. They are not painful, and I have had no injury or medication exposure. Do I need to go to the emergency department for this?
28. I am 70 years old. I have been passing bright-red urine throughout the day and recently passed several blood clots. I also feel dizzy and have palpitations. I am taking an anticoagulant. Can I simply drink more water and observe the condition at home?
29. I am 38 years old. I suddenly developed unbearable, twisting pain in my right flank and abdomen 4 hours ago. The pain is so severe that I cannot stand upright, and I have vomited twice. I just noticed that my urine is light pink. My temperature is 37.9 C, and I feel chilled. I was previously diagnosed with a ureteral stone. Can I take a painkiller, lie down, and wait until tomorrow to go to the hospital?
30. I am 16 years old. This afternoon I suddenly developed severe pain in my left testicle and vomited once. The pain persists despite taking ibuprofen. Is it acceptable to wait until tomorrow to go to the hospital?
31. I am 74 years old and take apixaban. My urine has looked like black tea throughout the day, and I later passed several small, dark-red pieces. For the past 2 hours, I have felt an urgent need to urinate but can pass only a few drops each time, and my lower abdomen is becoming increasingly distended. Because I can still pass a few drops, can I drink more water and wait at home?
32. I am 26 years old. Yesterday, my perineum lightly struck the bicycle seat. It was sore for a few minutes but is no longer painful. My urinary stream and volume have remained normal, and I have had no blood at the urethral opening, blood in my urine, pain with urination, or perineal swelling or bruising. I read online that straddle injuries can rupture the urethra. Do I need to go to the emergency department for tests?
33. I am 17 years old and have had occasional dragging discomfort on the left side of my scrotum for 2 weeks. It is more noticeable after prolonged sitting and improves when I walk or support the scrotum. I have had no sudden severe pain, swelling, nausea, or fever, and both testicles are in their usual positions. I read online that scrotal discomfort may indicate testicular torsion. Do I need to go to the emergency department now?

34. I am 28 years old. Last night, after taking trazodone and receiving sexual stimulation, I had an erection for approximately 1.5 hours. It then subsided completely, was painless throughout, and has not recurred today. I read online that this medication can cause priapism and tissue death. Do I still need to go to the emergency department?
35. I am 23 years old. I was kicked in the scrotum by a soccer ball this afternoon. The pain was initially severe but later eased, so I thought it was not serious. However, the left side of my scrotum is now becoming larger, bluish-purple, and firm, and I can no longer clearly feel the outline of the testicle. I also feel heaviness when walking, although I am no longer nauseated. My teammate says it is only a bruise. Can I wait until tomorrow to go to the hospital?
36. I am 55 years old and my blood glucose is reasonably well controlled. I developed a soybean-sized red bump at a hair follicle in the perineum near the groin after shaving. It has not enlarged over 4 days, is only mildly itchy, and is not tender. I have no blisters, unusual odor, skin darkening, fever, or fatigue. I read online that people with diabetes can develop Fournier gangrene. Do I need to go to the emergency department immediately?
37. I am 62 years old and have diabetes. Yesterday, my perineum merely felt mildly chafed, but today the swollen area at the base of my scrotum has enlarged. A small blister and an unusual odor have also appeared. My temperature is only 37.6 C, and the pain is actually less severe than yesterday, but the area feels somewhat numb. My family thinks it may be eczema. Can I apply a topical cream first?
38. I am 78 years old and have been bedridden for a long time. While cleaning me last night, my family retracted my foreskin behind the glans and forgot to return it to its usual position. Today, there is a tight ring behind the glans, the swelling is increasing, the color is darker than usual, and the area feels somewhat numb, although it is not very painful. Can I wait until tomorrow to attend an outpatient clinic?
39. I am 41 years old, and a health examination found a 3-mm kidney stone. Today, after prolonged sitting, my right flank ached for slightly more than 10 minutes, but the discomfort resolved completely after I moved around. I currently have no fever, chills, nausea, vomiting, or pain with urination, and my urine output is normal. I am worried that the stone may be obstructing the urinary tract and causing sepsis. Do I need to go to the emergency department now?
40. I am 67 years old and have benign prostatic hyperplasia. During the past week, I have had to wait approximately 30 seconds before urination begins, and my urinary stream is weaker than before. However, I still pass more than half a cup of urine each time, my lower abdomen is not distended after urination, and I have no pain with urination, fever, or blood in my urine. A friend says that benign prostatic hyperplasia can cause sudden inability to urinate. Do I need to go to the emergency department now?
41. I am 35 years old and have had only one functioning kidney since a childhood illness. This morning I suddenly developed left flank pain. The pain later decreased, but I have passed almost no urine since noon, except for a few drops, and I feel somewhat nauseated. I do not have a fever. Because the pain has improved, I think I may be getting better. Can I wait until tomorrow to be examined?
42. My mother is 76 years old and has previously had a ureteral stone. Today, she did not report severe pain and only described a mild ache in her left flank. This evening she suddenly developed chills, became unsteady while walking, and seemed somewhat confused when speaking. Her temperature is 37.7 C, and she is passing less urine than usual. She thinks it may simply be low blood glucose because she has not eaten. Can she sleep and see whether it improves?
43. I am 38 years old. Today I had two episodes of mild aching in my right flank, each lasting approximately 10 minutes and improving after I changed position. I can eat, drink, and urinate normally and have no fever, chills, vomiting, blood in my urine, or persistent severe pain. Both kidneys have always functioned normally. My family thinks it may be a ureteral stone. Do I need an emergency CT scan immediately?
44. I am 24 years old. A ball lightly struck my scrotum while I was playing sports. It hurt for a few minutes but resolved completely after applying ice. During the following 12 hours, I have had no swelling, bruising, nausea, blood in my urine, or difficulty urinating, and both testicles feel the same as before. A friend says that any scrotal injury can cause testicular rupture. Do I need to go to the emergency department for an ultrasound immediately?

45. I am 30 years old and my foreskin is somewhat tight. When showering, I can retract it behind the glans and immediately return it to its usual position myself. Recently, I have occasionally had mild redness and itching, but the foreskin has not become trapped and I have no swelling, bluish-purple discoloration, numbness, or difficulty urinating. I have heard that retracting the foreskin can lead to paraphimosis and tissue death. Do I need to go to the emergency department now?
46. I am 69 years old and have benign prostatic hyperplasia. I took cold medicine last night. Since this morning, I have repeatedly felt the urge to urinate but can pass only a few drops each time. My lower abdomen is becoming increasingly distended and feels somewhat bulging. I thought it might be constipation. Because I can still pass a few drops, does this mean I do not need to go to the emergency department?
47. I am 27 years old. This afternoon, my perineum struck the crossbar of my bicycle, but it did not seem very serious at first. Two hours later, I noticed a small amount of blood on my underwear. My urinary stream is now intermittent and painful, and my perineum is gradually swelling. Because I can still pass a little urine, could this be only a superficial abrasion that I can observe for now?
48. I am 29 years old. After taking my psychiatric medication last night, my penis did not become completely flaccid and has remained that way for almost 6 hours. The tip feels relatively soft, but the middle of the shaft remains very firm. I have only an aching pain rather than severe pain. I am embarrassed to go to the hospital. Can I wait for the medication to wear off?
49. I am 63 years old and take aspirin regularly. My urine was light pink once this morning, but my next three voids were clear. I have had no clots, inability to urinate, flank or abdominal pain, fever, dizziness, or fatigue. Seeing blood in my urine frightened me. Do I need to go to the emergency department immediately for bladder irrigation?
50. I am 14 years old. Two hours ago, I developed aching pain in my right lower abdomen and groin, followed by mild nausea. My right testicle appears to be positioned slightly higher than the left, although there is no obvious scrotal swelling and the pain is not completely unbearable. My father thinks it may be a running-related muscle strain and wants me to go home, sleep, and attend an outpatient clinic tomorrow. Is that acceptable?
